# A meta-analysis of *Lactobacillus*-based probiotics for growth performance and intestinal morphology in piglets

**DOI:** 10.3389/fvets.2022.1045965

**Published:** 2022-11-08

**Authors:** Cuipeng Zhu, Jiacheng Yao, Miaonan Zhu, Chuyang Zhu, Long Yuan, Zhaojian Li, Demin Cai, Shihao Chen, Ping Hu, Hao-Yu Liu

**Affiliations:** ^1^College of Animal Science and Technology, Yangzhou University, Yangzhou, China; ^2^Institute of Epigenetics and Epigenomics, Yangzhou University, Yangzhou, China

**Keywords:** *Lactobacillus*, probiotics, piglets, antibiotic alternatives, intestinal morphology, growth performance

## Abstract

Antibiotics are widely used as growth promoters (AGPs) in livestock production to improve animal performance and health. However, pig producers today face the prohibition of in-feed antimicrobials and have to find safe and effective alternatives. *Lactobacillus* species are active microorganisms that convey multiple beneficial effects to the host and are one of the most promising AGPs replacements. Here, we aim to comprehensively assess the effects of *Lactobacillus* spp. supplementation on growth performance and intestinal morphology (villus height [VH], crypt depth [CD], and the V/C ratio) of piglets. Among the 196 identified studies, 20 met the criteria and were included in the meta-analysis. The effects of *Lactobacillus*-based probiotics supplementation on growth performance and intestinal morphology were analyzed using a random-effects model. And the publication bias was evaluated by funnel plots. Our results revealed that *Lactobacillus* spp. supplementation significantly improved the growth performance, including average daily feed intake (ADFI), average daily gain (ADG), and the gain-to-feed ratio (G/F) in piglets (*P* < 0.05). Meanwhile, *Lactobacillus* spp. remarkably increased VH and the V/C ratio (*P* < 0.05) in the small intestine, including the duodenum, jejunum, and ileum, which might contribute to an improved digestive capacity of these animals. In conclusion, our findings provide concrete evidence of the growth-promoting effects of *Lactobacillus* spp. supplementation in piglets and a better understanding of the potential of *Lactobacillus*-based probiotics as AGPs alternatives in pig production.

## Introduction

Antibiotics used as growth promoters at sub-therapeutic doses to animals are an integral part of livestock production. Large-scale addition of antibiotic growth promoters (AGPs) to animal feed can help to improve production efficiency by improving animal performance represented by average daily feed intake (ADFI), average daily gain (ADG), and gain-to-feed ratio (G/F) (1–3). Moreover, AGPs may alter intestinal morphology and promote digestion and absorption of nutrients in the intestine, which also contribute to animal growth and development (4). In pigs, the improved performance attributed to AGPs addition was between 4 to 8% (3, 5). However, the overuse of antibiotics has induced the development of multi-drug-resistant microorganisms in farm animals. It could seriously endanger animal production and public health (6, 7). Due to this concern, antibiotics used as growth promoters in livestock have been banned in the European Union since 2006 (8). As of 2017, the US has banned the use of medically-important antimicrobials for preventative and growth-promotion purposes the in the livestock sector (9). In addition, China prohibited in-feed antimicrobials in animal production in 2020 (10). Several antibiotic alternatives have been developed, studied, and tested in livestock to face the increasing global restrictions on antibiotic usage while maintaining animal health and performance.

Probiotics are live microbial supplements in adequacy or components of bacteria that confer beneficial effects on the intestinal health of the host (11) through occupying binding sites of the intestinal mucosa or competing for nutrients and niches with pathogenic bacteria (12, 13). Numerous studies have revealed that *Lactobacillus* species improve the growth performance and decrease the diarrhea ratio of piglets by enhancing nutrition digestibility and intestinal barrier function (14–19). Among the tested probiotics, *Lactobacillus* species are considered one of the most promising replacements and therefore represent a safe opportunity to substitute AGPs in pigs (18, 20). However, the wide variety of *Lactobacillus* species and different experimental designs make it difficult to comprehensively understand and further evaluate the effects of *Lactobacillus* species on swine and finally use them to replace AGPs at large in production. In this regard, meta-analysis constitutes a method integrating and analyzing numerous independent studies on the same subjects and makes the most representative conclusions (21). More importantly, in a meta-analysis, as the amount of data used increases, the precision of estimates can be improved on separate studies with different results (22). It justifies our attempt to employ this approach to determine the effects of *Lactobacillus*-based probiotics in pigs in the background of the emerging antibiotics alternative research. To provide a mechanism for estimating the effect degree, a strict design and clear selection criteria of studies, and the measurement index are necessary (22).

It is worth mentioning that the gut development of piglets is sensitive to alterations of feed components, reflected by their morphological changes (23). Histologically, the porcine intestine follows the general structure throughout its length and is similar to other monogastric animals and humans: the mucosa surface is covered by a monolayer of epithelium including absorptive enterocytes, goblet, and endocrine cells, *etc*. (23). The surface lining of epithelium quickly renews themselves and contributes to the absorptive surface and capacity of the intestine. It is commonly accepted to determine villus height, crypt depth, and the V/C ratio as “gold standards” of intestinal morphology, while these histological parameters could be used as a tool to evaluate gut function and responses toward feed ingredients (24). In the current study, we therefore performed a set of meta-analyses to delineate the effects of *Lactobacillus* species on pig growth performance and intestinal morphology.

## Materials and methods

### Study search and inclusion criteria

The protocols used were following the MOOSE guidelines (25). This study aimed to analyze the effects of *Lactobacillus* species supplementation in piglets with or without *E. coli*/LPS challenges on growth performance and intestinal morphological parameters. We have identified studies using *Lactobacillus* spp. including *Lactobacillus delbrueckii, Lactobacillus reuteri, Lactobacillus plantarum* and *Lactobacillus acidophilus etc*. published in English from June 2010 to June 2022. The search strategy consisted of a search of English databases, including PubMed, Google scholar, Cochrane library, semantic scholar Embased and Clinical Trials, and a search of Chinese databases, including VIP, CNKI, and WANFANG Data. The search terms included: (*Lactobacillus* OR *Lactobacilli* OR Lactic acid bacteria) AND (piglets OR piggy OR pigling) AND (growth performance OR average daily gain OR average daily feed intake OR feed efficiency) AND (duodenal villus height, crypt depth, and villus height to crypt depth OR jejunal villus height, crypt depth and villus height to crypt depth OR Ileal villus height, crypt depth and villus height to crypt depth) in titles or abstracts.

The eligibility for inclusion of all studies identified from the searches was independently assessed and compared by the authors where the inclusion/exclusion criteria described previously were also considered (26). Manual selections were conducted on all returned publications based on the relevance of the titles and/or abstracts of the publications to *Lactobacillus*. Prerequisites of the selected publications were a downloadable full text and available data in English regarding *Lactobacillus*-based probiotics for growth performance and intestinal morphology in piglets.

### Exclusion criteria

**Figure 1:**
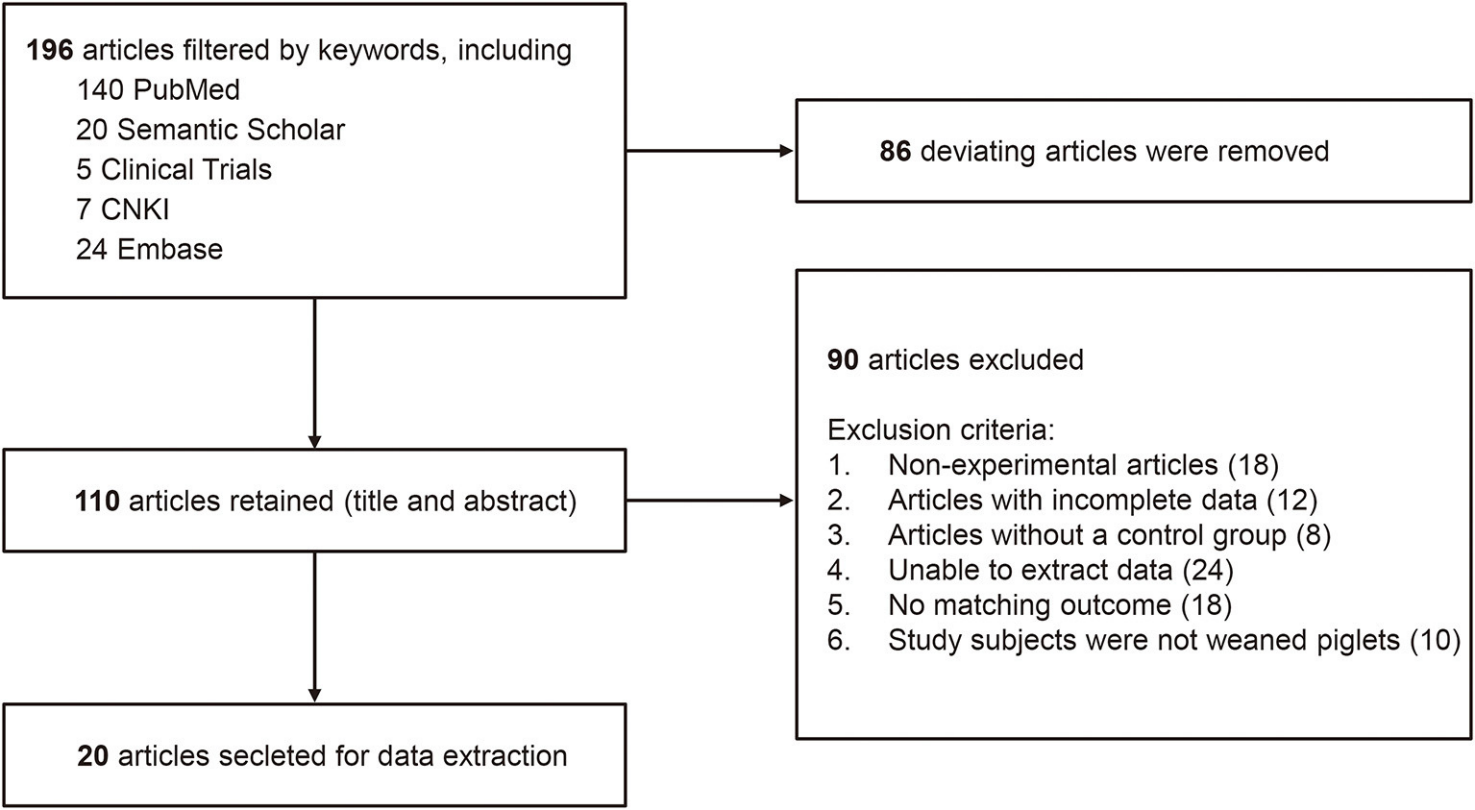
A flow diagram of studies included in the meta-analysis *Lactobacillus*-based probiotics for growth performance and intestinal physiology in piglets.

Studies excluded from this systematic review and meta-analysis were those that met the following criteria. Firstly, non-experimental articles (review articles); Secondly, articles with incorrect or incomplete data; Thirdly, articles without a control group; Finally, non-probiotics added or combined with other drugs and preparations in the experimental groups. A flow diagram was used to summarize the process of the article selection and study inclusion/exclusion for our meta-analysis (Figure 1).

### Data extraction

Data relating to the effects of *Lactobacillus* spp. supplementation with or without *E. coli*/LPS challenges on the growth performance and intestinal physiology in piglets were collected from each selected article using a custom-tailored excel form that included detailed information as follows: animals (breed, sex distribution, and age), sample size, experimental design including the control group set up, target *Lactobacillus* strains, the amount (dose) of probiotic supplementation, administration methods and duration, authors, When results were available only in graphical format, data were extracted using Review Manager (Version 5.4 provided by Cochrane Training). Graph digitization has been previously shown to be a valid method for extracting study data (27). If the data were unclear or some key data were missing, we attempted to contact the corresponding authors through email to obtain further information. The outcomes were as follows: ADG (average daily gain); ADFI (average daily feed intake); G/F (gain-to-feed ratio); diarrhea rate; Duodenum VH (villus height), CD (crypt depth) and V/C (villus height: crypt depth); Jejunum VH, CD and V/C; and Ileum VH, CD and V/C.

In our initial search, 2123 English language records were hit which, after screening, revealed 196 unique articles. A total of 20 papers that fulfilled the selection criteria were included in the quantitative meta-analysis, including Suo et al. (28); Sayan et al. (14); Chen et al. (29); Liu et al. (30); Yi et al. (15); Yang et al. (31); Wang et al. (32); Qiao et al. (33); Lan et al. (34); Lee et al. (35); Li et al. (36); Tang et al. (37); Tian et al. (38); Sonia et al. (39); Eliette et al. (40); Liu et al. (41); Moturi et al. (42); Lee et al. (43); Cao et al. (44); and Jeong et al. (45). The characteristics of each study are shown in Table 1. The median initial body weight (BW) of the piglet was 6.50 kg (from 1.50 kg to 15.6 kg). In total, 6 studies included used antibiotics as the control group, and 8 studies included had piglets challenged with *E. coli* or LPS.

**Table 1:** Information of included studies on the effects of *Lactobacillus*-based probiotics in pigs^a^.

**Studies**	**Period**	**Treatment**	**Added amount**	**Control**	**Sample size and sex**	**BW ranges**	**Outcomes**	**Challenge**
Suo et al. (28)	post–weaning	*L. plantarum*	1 × 10^9^ CFU/d	Basal diet and antibiotics	150 NA	7.69–11.59 kg	ADG, ADFI, G/F, duodenum V/C, ileum V/C, jejunum V/C	NA
Sayan et al. (14)	pre–weaning	*L. salivarius*	1 × 10^9^ CFU/mL	Basal diet	201 NA	1.59–6.18 kg	ADG, ADFI, G/F, ileum V/C	*E. coli*
Chen et al. (29)	post–weaning	*L. delbrueckii*	2.01 × 10^10^ CFU/g	Basal diet	36 average	7.79–16.15 kg	ADG, ADFI, G/F, duodenum V/C, ileum V/C, jejunum V/C	LPS
Liu et al. (30)	pre–weaning	*L. fermentum*	6 × 10^9^ CFU/mL	Basal diet	36 NA	2.31–4.73 kg	ADG, jejunum V/C	NA
Yi et al. (15)	post–weaning	*L. reuteri*	5 × 10^10^ CFU/kg	Basal diet and antibiotics	144 average	6.49–9.89 kg	ADG, ADFI, G/F, duodenum V/C, ileum V/C, jejunum V/C	NA
Yang et al. (31)	pre–weaning	*L. plantarum*	5 × 10^10^ CFU/kg	Basal diet	72 males	2.41–7.06 kg	ADG, ADFI, G/F, duodenum V/C, ileum V/C, jejunum V/C	*E. coli*
Wang et al. (32)	pre–weaning	*L. plantarum*	1 × 10^10^ CFU/d	Basal diet	60 NA	1.50–3.56 kg	duodenum V/C, ileum V/C, jejunum V/C	NA
Qiao et al. (33)	post–weaning	*L. acidophilus*	0.05%, 0.1%, 0.2%	Basal diet and antibiotics	150 females	7.53–16.31 kg	ADG, ADFI, G/F	LPS
Lan et al. (34)	post–weaning	*L. acidophilus*	1, 2, 3 g/kg	Basal diet and antibiotics	175 NA	7.15 kg–NA	ADG, ADFI, G/F	NA
Lee et al. (35)	post–weaning	*L. plantarum*	10^8^, 10^9^, 10^10^ CFU/kg	Basal diet and antibiotics	108 NA	8.74–21.84 kg	ADG, ADFI, G/F	*E. coli*
Li et al. (36)	post–weaning	*L. mucosae*	1 × 10^9^ CFU/mL	Basal diet	104 average	5.90–18.82 kg	ADG, ADFI, G/F, duodenum V/C, ileum V/C, jejunum V/C	*E. coli*, LPS
Tang et al. (37)	post–weaning	*L. reuteri*	5 × 10^10^ CFU/kg	Basal diet	90 NA	6.1–14.9 kg	ADG, ADFI, G/F, ileum V/C, jejunum V/C, duodenum V/C	NA
Tian et al. (38)	post–weaning	*L. reuteri*	5 × 10^10^ CPU/kg	Basal diet	144 males	6.49 kg–NA	ADG, ADFI, F/G, ileum V/C, jejunum V/C, duodenum V/C	NA
Tabasum et al. (39)	post–weaning	*L. reuteri*	0.5%, 0.04%	Basal diet and antibiotics	96 NA	8.00 kg–NA	ADG, ADFI, G/F	*E. coli*
Riboulet-Bisson et al. (40)	pre–weaning	*L. salivarius*	1 × 10^8^ CFU/mL	Basal diet	30 NA	12.7–34.4 kg	ADG, ADFI, G/F	NA
Liu et al. (41)	post–weaning	*L. brevis*	0.4, 0.8 g/kg	Basal diet	144 NA	15.6–24.9 kg	ADG, ADFI, G/F	NA
Moturi et al. (42)	nursery	*L. salivarus*	1 × 10^8^ CFU/mL	Basal diet	30 NA	1.54–6.22 kg	ADG, ileum V/C, jejunum V/C, duodenum V/C	NA
Lee et al. (43)	post–weaning	*L. acidophilus*	0.10%	Basal diet	40 NA	7.10–11.60 kg	ADG, ADFI, G/F	LPS
Cao et al. (44)	post–weaning	*L. acidophilus*	1 × 10^8^ CFU/mL	Basal diet	180 NA	6.2 kg–NA	ADG, ADFI, G/F, jejunum V/C	NA
Jeong et al. (45)	post–weaning	*L. casei*	1 × 10^11^ CFU/mL	Basal diet	240 NA	7.05 kg–NA	ADG, ADFI, G/F, ileum V/C, jejunum V/C, duodenum V/C	NA

a BW, body weight; ADG, average daily gain; ADFI, average daily feed intake; G/F, gain: feed ratio; NA, not applicable; LPS, lipopolysaccharide; V/C, villus height, crypt depth ratio.

### Statistical analyses

Considering that there are contrary data within the range of the research purpose and the heterogeneity between different studies may interfere with the analyzed results, we used a random-effects model to compute the 95% confidence interval (95% CI) of the standardized mean difference (SMD). Heterogeneity was assessed using chi-square test and the I^2^ parameter (30–60% indicating moderate, 50–90% indicating substantial, and 75–100% indicating considerable heterogeneity). For the identification and assessment of reporting bias, we tested funnel plot symmetry by the Begg and Egger method (47). All the above analyses were performed by the Review Manager (Version 5.4 provided by Cochrane Training). *P* < 0.05 is considered significant.

## Results

### Forest plots and sensitivity analysis of *Lactobacillus* species to growth performance in piglets

**Figure 2:**
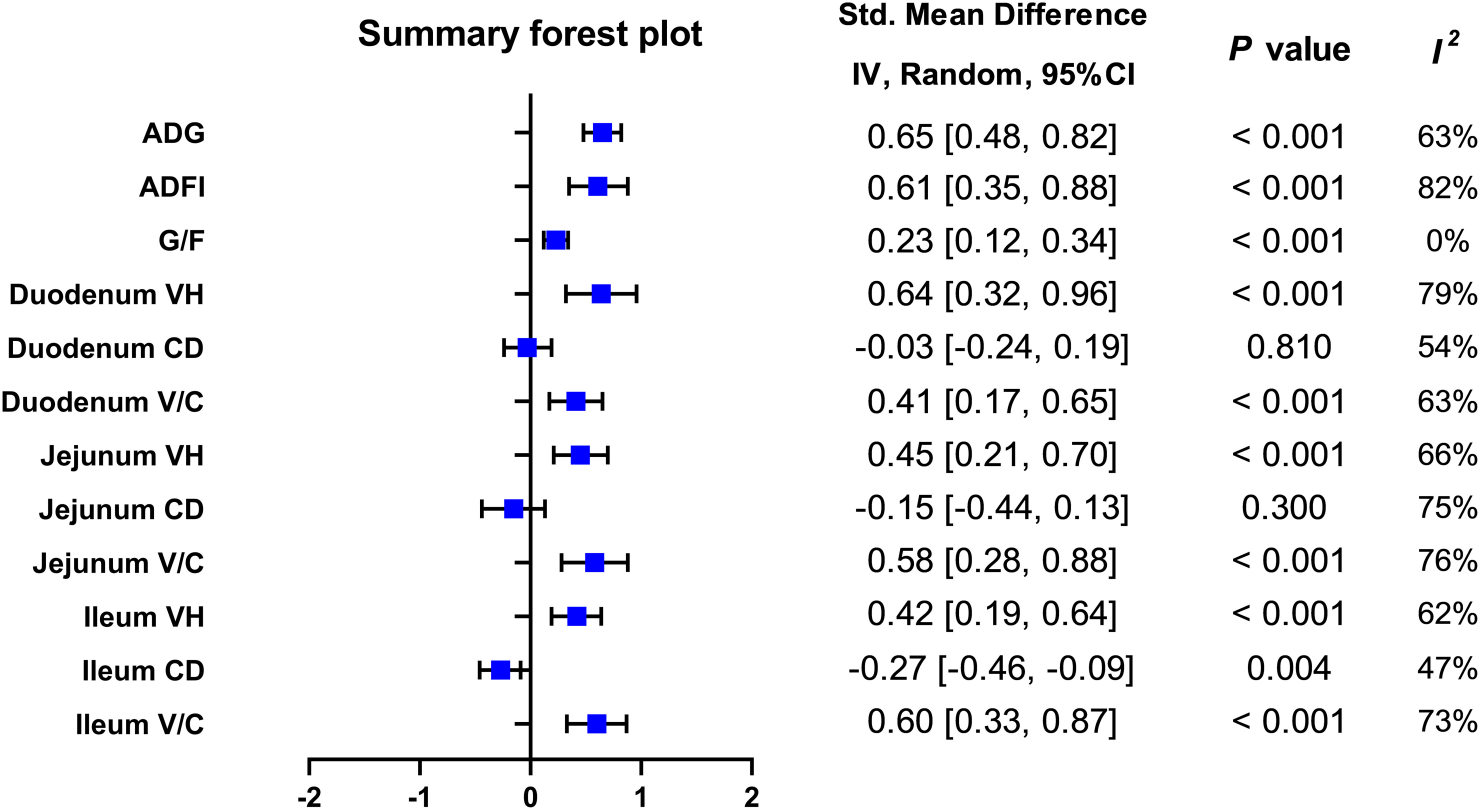
Summary forest plot of included outcomes on pigs. ADG, average daily gain; ADFI, average daily feed intake; CI, confidence interval; G/F, gain: feed ratio; V/C, villus height: crypt depth ratio.

**Figure 3:**
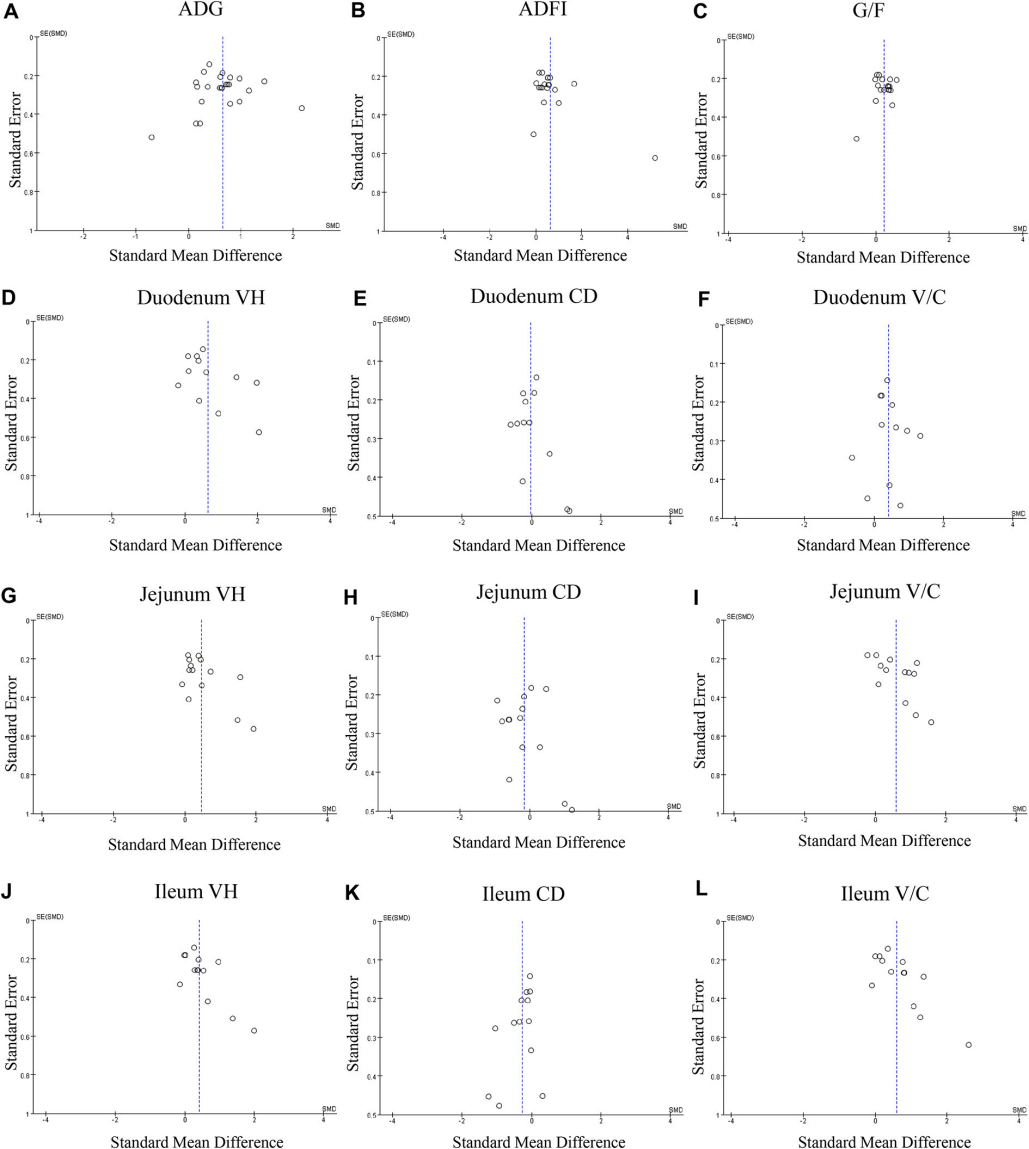
Funnel plots of the publication bias analysis of included outcomes on pigs. **(A)** Average daily gain, **(B)** Average daily feed intake. **(C)** Gain: feed ratio. **(D)** Duodenal VH. **(E)** Duodenal CD. **(F)** duodenal V/C. **(G)** Jejunal VH. **(H)** Jejunal CD. **(I)** Jejunal. **(J)** Ileal VH. **(K)** Ileal CD. **(L)** Ileal V/C. *n* = 12; CD, crypt depth; SE, standard error; SMD, standard mean difference; V/C, villus height: crypt depth; VH, villus height; V/C, villus height: crypt depth.

A meta-analysis was performed to examine the effect of probiotic *Lactobacillus* spp. on ADG (24 trials, *n* = 1683 subjects). The summarized results of standardized mean difference (SMD) and 95% confidence interval (CI) for each study are shown in Table 2. There was a significant positive correlation between ADG and the *Lactobacillus* spp. addition (SMD = 0.65; 95% CI: 0.48~0.82; I^2^ = 63%; *P* < 0.001), and *Lactobacillus* spp. supplementation improved the ADG of piglets. Next, the effects of *Lactobacillus* spp. supplementation on ADFI in piglets were determined by forest plots and sensitivity analysis (19 trials, *n* = 1346 subjects). The summarized results of SMD and 95% CI are shown in Table 3. *Lactobacillus* spp. remarkably increased the ADFI of piglets compared to the control group (SMD = 0.61; 95% CI: 0.35~0.88; *P* < 0.001), but showed a high heterogeneity (I^2^ = 82%), indicating a high degree of inter-study variability. Furthermore, we analyzed the effects of probiotic *Lactobacillus* spp. on the G/F ratio (18 trials, *n* = 1298 subjects) including forest plots and sensitivity analysis. The summarized results are shown in Table 4, the resulting forest map is shown in Figure 2 and the publication bias analysis of all parameters is shown in Figures 3A–L. We found that G/F was significantly increased after *Lactobacillus* spp. supplementation (SMD = 0.23; 95% CI: 0.12~0.34; I^2^ = 0%; *P* < 0.001).

**Table 2:** Effects of *Lactobacillus*–based probiotics on the average daily gain (ADG) of pigs from included studies.

**Studies**	**Treatment**	**Added amount**	**Experimental**			**Control**			**Weight**	**Std. mean difference**
			**Mean**	**SD**	**Total**	**Mean**	**SD**	**Total**		**IV, Random, 95%CI**
Suo et al. (28)	*L. plantarum*	1 × 10^9^ CFU/d	469	57.44	30	455	101.05	30	4.30%	0.17 [−0.34, 0.68]
Riboulet-Bisson et al. (40)	*L. salivarius*	1 × 10^8^ CFU/mL	748	55.03	8	798	78.00	8	2.00%	−0.70 [−1.72, 0.32]
Liu et al. (46)	*L. brevis*	0.4 g/kg	361	116.92	48	246	116.92	48	4.90%	0.98 [0.55, 1.40]
Liu et al. (46)	*L. brevis*	0.8 g/kg	318	116.92	48	246	116.92	48	5.00%	0.61 [0.20, 1.02]
Sayan et al. (14)	*L. salivarius*	1 × 10^9^ CFU/mL	191	64.26	87	163	73.46	114	5.90%	0.40 [0.12, 0.68]
Yi et al. (15)	*L. reuteri*	5 × 10^10^ CFU/kg	243	56.01	48	198	56.01	48	5.00%	0.80 [0.38, 1.21]
Liu et al. (17)	*L. fermentum*	6 × 10^9^ CFU/mL	184	26.19	18	148	56.75	18	3.30%	0.80 [0.11, 1.48]
Qiao et al. (33)	*L. acidophilus*	0.05%	295	74.31	30	267	74.31	30	4.30%	0.37 [−0.14, 0.88]
Qiao et al. (33)	*L. acidophilus*	0.10%	312	74.31	30	267	74.31	30	4.30%	0.60 [0.08, 1.12]
Qiao et al. (33)	*L. acidophilus*	0.20%	314	74.31	30	267	74.31	30	4.30%	0.62 [0.11, 1.14]
Moturi et al. (42)	*L. salivarus* 144	1 × 10^8^ CFU/ mL	223	12.83	10	221	12.83	10	2.50%	0.15 [−0.73, 1.03]
Moturi et al. (42)	*L. salivarus* 160	1 × 10^8^ CFU/ mL	224	12.83	10	221	12.83	10	2.40%	0.22 [−0.66, 1.10]
Yang et al. (31)	*L. plantarum*	5 × 10^10^ CFU/kg	270	65.48	18	253	65.48	18	3.40%	0.25 [−0.40, 0.91]
Tang et al. (37)	*L. plantarum*	5 × 10^10^ CFU/kg	244	57.38	30	207	57.38	30	4.30%	0.64 [0.12, 1.16]
Tang et al. (37)	*L. reuteri*	5 × 10^10^ CFU/kg	274	57.38	30	207	57.38	30	4.10%	1.15 [0.60, 1.70]
Lan et al. (34)	*L. acidophilus*	1g/kg	488	60.02	35	445	60.02	35	4.50%	0.71 [0.22, 1.19]
Lan et al. (34)	*L. acidophilus*	2g/kg	490	60.02	35	445	60.02	35	4.50%	0.74 [0.26, 1.23]
Lan et al. (34)	*L. acidophilus*	3g/kg	492	60.02	35	445	60.02	35	4.50%	0.77 [0.29, 1.26]
Lee et al. (43)	*L. acidophilus*	0.10%	328	12.11	20	316	12.11	20	3.40%	0.97 [0.31, 1.63]
Cao et al. (44)	*L. acidophilus*	1 × 10^8^ CFU/mL	275	40.71	36	269	40.71	36	4.60%	0.15 [−0.32, 0.61]
Tabasum et al. (39)	*Lactobacillus*	0.50%	390	66.86	24	243	66.86	24	3.10%	2.16 [1.44, 2.89]
Jeong et al. (45)	*L. casei*	0.10%	322	47.65	60	291	47.65	60	5.30%	0.65 [0.28, 1.01]
Jeong et al. (45)	*L. casei*	0.20%	325	47.65	60	311	47.65	60	5.40%	0.29 [−0.07, 0.65]
Tian et al. (38)	*L. reuteri*	5 × 10^10^ CFU/kg	675	15.87	48	650	18.39	48	4.70%	1.44 [0.99, 1.90]
Total (95% CI)					828			855	100.00%	0.65 [0.48, 0.82]

Heterogeneity, Tau^2^ = 0.11; Chi^2^ = 62.39, df = 23 (P < 0.0001); I^2^ = 63%.

Test for overall effect, Z = 7.45 (P < 0.00001).

**Table 3:** Effects of *Lactobacillus*–based probiotics on the average daily feed intake (ADFI) pigs from included studies.

**Studies**	**Treatment**	**Added amount**	**Experimental**			**Control**			**Weight**	**Std. mean difference**
			**Mean**	**SD**	**Total**	**Mean**	**SD**	**Total**		**IV, Random, 95%CI**
Riboulet-Bisson et al. (40)	*L. salivarius*	1 × 10^8^ CFU/mL	962	105.82	8	973	91.01	8	3.50%	−0.11 [−1.09, 0.88]
Liu et al. (46)	*L. brevis*	0.4 g/kg	679	163.13	48	591	163.13	48	5.80%	0.54 [0.13, 0.94]
Liu et al. (46)	*L. brevis*	0.8 g/kg	678	163.13	48	591	163.13	48	5.80%	0.53 [0.12, 0.94]
Yi et al. (15)	*L. reuteri*	5 × 10^10^ CFU/kg	358	77.02	48	310	77.02	48	5.80%	0.62 [0.21, 1.03]
Qiao et al. (33)	*L. acidophilus*	0.05%	530	76.89	30	519	76.89	30	5.40%	0.14 [−0.37, 0.65]
Qiao et al. (33)	*L. acidophilus*	0.10%	536	76.89	30	519	76.89	30	5.40%	0.22 [−0.29, 0.73]
Qiao et al. (33)	*L. acidophilus*	0.20%	541	76.89	30	519	76.89	30	5.40%	0.28 [−0.23, 0.79]
Yang et al. (31)	*L. plantarum*	5 × 10^10^ CFU/kg	234	21.83	18	226	21.83	18	4.80%	0.36 [−0.30, 1.02]
Tang et al. (37)	*L. plantarum*	5 × 10^10^ CFU/kg	358	74.65	30	321	74.65	30	5.40%	0.49 [−0.02, 1.00]
Tang et al. (37)	*L. reuteri*	5 × 10^10^ CFU/kg	384	74.65	30	321	74.65	30	5.30%	0.83 [0.30, 1.36]
Lan et al. (34)	*L. acidophilus*	1g/kg	682	48.02	35	663	48.02	35	5.60%	0.39 [−0.08, 0.86]
Lan et al. (34)	*L. acidophilus*	2g/kg	690	48.02	35	663	48.02	35	5.50%	0.56 [0.08, 1.03]
Lan et al. (34)	*L. acidophilus*	3g/kg	691	48.02	35	663	48.02	35	5.50%	0.58 [0.10, 1.06]
Lee et al. (43)	*L. acidophilus*	0.10%	405	14.82	20	390	14.82	20	4.80%	0.99 [0.33, 1.65]
Cao et al. (44)	*L. acidophilus*	1 × 10^8^ CFU/mL	369	51.66	36	368	51.66	36	5.60%	0.02 [−0.44, 0.48]
Tabasum et al. (39)	*Lactobacillus*	0.50%	695	40.09	24	484	40.09	24	2.80%	5.18 [3.96, 6.40]
Jeong et al. (45)	*L. casei*	0.10%	461	93.74	60	433	93.74	60	6.00%	0.30 [−0.06, 0.66]
Jeong et al. (45)	*L. casei*	0.20%	453	93.74	60	439	93.74	60	6.00%	0.15 [−0.21, 0.51]
Tian et al. (38)	*L. reuteri*	5 × 10^10^ CFU/kg	1770	50.25	48	1685	50.22	48	5.60%	1.68 [1.21, 2.15]
Total (95% CI)					673			673	100.00%	0.61 [0.35, 0.88]

Heterogeneity, Tau^2^ = 0.28; Chi^2^ = 99.69, df = 18 (P < 0.00001); I^2^ = 82%.

Test for overall effect, Z = 4.49 (P < 0.00001).

**Table 4:** Effects of *Lactobacillus*–based probiotics on the gain to feed (G/F) ratio of pigs from included studies.

**Studies**	**Treatment**	**Added amount**	**Experimental**			**Control**			**Weight**	**Std. mean difference**
			**Mean**	**SD**	**Total**	**Mean**	**SD**	**Total**		**IV, Random, 95%CI**
Riboulet-Bisson et al. (40)	*L.salivarius*	1 × 10^8^ CFU/mL	0.78	0.09	8	0.82	0.05	8	1.20%	−0.52 [−1.52, 0.48]
Liu et al. (46)	*L. brevis*	0.4 g/kg	0.53	0.29	48	0.417	0.29	48	7.30%	0.39 [−0.01, 0.80]
Liu et al. (46)	*L. brevis*	0.8 g/kg	0.47	0.29	48	0.417	0.29	48	7.50%	0.18 [−0.22, 0.58]
Yi et al. (15)	*L. reuteri*	5 × 10^10^ CFU/kg	0.68	0.07	48	0.64	0.07	48	7.20%	0.57 [0.16, 0.98]
Qiao et al. (33)	*L. acidophilus*	0.05%	0.56	0.18	30	0.518	0.18	30	4.60%	0.22 [−0.29, 0.73]
Qiao et al. (33)	*L. acidophilus*	0.10%	0.59	0.18	30	0.518	0.18	30	4.60%	0.36 [−0.15, 0.87]
Qiao et al. (33)	*L. acidophilus*	0.20%	0.58	0.18	30	0.518	0.18	30	4.60%	0.34 [−0.17, 0.85]
Yang et al. (31)	*L. plantarum*	5 × 10^10^ CFU/kg	1.16	0.09	18	1.12	0.09	18	2.70%	0.45 [−0.21, 1.11]
Tang et al. (37)	*L. plantarum*	5 × 10^10^ CFU/kg	0.68	0.12	30	0.664	0.12	30	4.70%	0.14 [−0.37, 0.64]
Tang et al. (37)	*L. reuteri*	5 × 10^10^ CFU/kg	0.71	0.12	30	0.664	0.12	30	4.60%	0.40 [−0.11, 0.92]
Lan et al. (34)	*L. acidophilus*	1g/kg	0.72	0.12	35	0.671	0.12	35	5.40%	0.37 [−0.10, 0.84]
Lan et al. (34)	*L. acidophilus*	2g/kg	0.71	0.12	35	0.671	0.12	35	5.40%	0.34 [−0.13, 0.81]
Lan et al. (34)	*L. acidophilus*	3g/kg	0.71	0.12	35	0.671	0.12	35	5.40%	0.33 [−0.14, 0.80]
Lee et al. (43)	*L. acidophilus*	0.10%	0.81	0.05	20	0.81	0.05	20	3.10%	0.00 [−0.62, 0.62]
Cao et al. (44)	*L. acidophilus*	1 × 10^8^ CFU/mL	0.75	0.30	36	0.73	0.30	36	5.60%	0.07 [−0.40, 0.53]
Jeong et al. (45)	*L. casei*	0.10%	0.7	0.31	60	0.67	0.31	60	9.30%	0.10 [−0.26, 0.45]
Jeong et al. (45)	*L. casei*	0.20%	0.72	0.31	60	0.71	0.31	60	9.40%	0.03 [−0.33, 0.39]
Tian et al. (38)	*L. reuteri*	5 × 10^10^ CFU/kg	0.38	0.14	48	0.386	0.28	48	7.50%	−0.02 [−0.42, 0.38]
Total (95% CI)					649			649	100.00%	0.23 [0.12, 0.34]

Heterogeneity: Tau^2^ = 0.00; Chi^2^ = 11.79, df = 17 (P = 0.81); I^2^ = 0%.

Test for overall effect: Z = 4.05 (P < 0.0001).

### Forest plots and sensitivity analysis of *Lactobacillus* species to the duodenal morphology in piglets

The *Lactobacillus*-promoted growth performance of piglets identified by our analysis prompted us to further study the effects of *Lactobacillus* spp. on parameters like villus height (VH), crypt depth (CD), and the V/C ratio, representing changes in the small intestinal morphology of piglets (Table 5, Figure 2). First, we found that porcine duodenal VH (12 trials, *n* = 877 subjects) was significantly increased by *Lactobacillus* spp. supplementation compared to the control (SMD = 0.64; 95% CI: 0.32~0.92; I^2^ = 79%; *P* < 0.001). In contrast, no significant correlation between the addition of *Lactobacillus* spp. and the duodenal CD was observed (12 trials, n = 877 subjects; SMD = −0.03; 95% CI:−0.24~0.19; I^2^ = 54%; *P* = 0.81). In the duodenum of piglets, the analysis showed a positive correlation between the V/C ratio and *Lactobacillus* spp. supplementation (SMD = 0.41; 95% CI: 0.17~0.65; I^2^ = 65%; *P* = 0.002).

**Table 5:** Effects of *Lactobacillus*–based probiotics on small intestinal morphology of pigs from included studies.

**Outcomes**	**Effect size**			***P*–value**	**Heterogeneity**			***P*–value**
	**No. of trials**	**SMD**	**95%Cl**		**I^2^**	**Tau^2^**	**Chi^2^**	
**Duodenum**								
Villus height (VH)	12	0.64	[0.32, 0.96]	<0.001	79%	0.23	52.18	<0.001
Crypt depth (CD)	12	−0.03	[−0.24, 0.19]	0.810	54%	0.07	23.83	0.010
VH/CD	12	0.41	[0.17, 0.65]	<0.001	63%	0.10	30.04	0.002
**Jejunum**								
Villus height (VH)	14	0.45	[0.21, 0.70]	<0.001	66%	0.13	38.07	<0.001
Crypt depth (CD)	14	−0.15	[−0.44, 0.13]	0.300	75%	0.21	53.03	<0.001
VH/CD	13	0.58	[0.28, 0.88]	<0.001	76%	0.22	50.84	<0.001
**Ileum**								
Villus height (VH)	13	0.42	[0.19, 0.64]	<0.001	62%	0.10	31.95	0.001
Crypt depth (CD)	13	−0.27	[−0.46, −0.09]	0.004	47%	0.05	22.77	0.030
VH/CD	13	0.60	[0.33, 0.87]	<0.001	73%	0.16	44.85	<0.001

### Forest plots, sensitivity analysis, and funnel plots of *Lactobacillus* species to jejunal morphology in piglets

In the porcine jejunum, the effects of probiotics on VH, CD, and the V/C ratio were evaluated (Table 5, Figure 2). In the jejunum of piglets (14 trials, *n* = 880 subjects), the analysis showed a positive effect with the addition of *Lactobacillus* spp. on the VH values (SMD = 0.45; 95% CI: 0.21~0.70; I^2^ = 65%; *P* = 0.005). In addition, the effect of *Lactobacillus* spp. addition on the jejunal CD was also examined (14 trials, *n* = 880 subjects), and *Lactobacillus* supplementation significantly decreased CD, compared to the control (SMD = −0.15; 95% CI: −0.44~0.13; I^2^ = 75%; *P* < 0.001). The changes of the V/C ratio between the control and the addition of *Lactobacillus* spp. were analyzed (13 trials, *n* = 844 subjects), and found that it was significantly increased by the addition of *Lactobacillus* spp. (SMD = 0.58; 95% CI: 0.28~0.88; I^2^ = 76%; *P* < 0.001).

### Forest plots and sensitivity analysis of *Lactobacillus* species to ileum morphology in piglets

Finally, we analyzed the effects of probiotic *Lactobacillus* spp. supplementation on the ileal morphology of piglets (13 trials, *n* = 973 subjects; Table 5, Figure 2). The ileal VH was strongly positively correlated with *Lactobacillus* spp. addition (SMD = 0.42; 95% CI: 0.19~0.64; I^2^ = 62%; *P* = 0.001). Next, the effects of *Lactobacillus* spp. addition on the ileal CD were determined (13 trials, *n* = 973 subjects), *Lactobacillus* spp. was associated with a significant reduction of the ileal CD (SMD = −0.27; 95% CI: −0.46~-0.09; I^2^ = 47%; *P* =0.03). For the ileal V/C ratio (13 trials, n = 973 subjects), the addition of probiotic *Lactobacillus* spp. significantly increased this value when compared to the control (SMD = 0.60; 95% CI: 0.33~0.87; I^2^ = 73%; *P* < 0.001).

## Discussion

Antibiotics are widely used as growth promoters in livestock to improve animal growth performance and health. Several hypotheses on the AGPs' mode of action have been proposed, including reducing pathogenic load and toxin production and inhibiting gut disorders while improving intestinal physiology (48, 49). However, due to the side effects (*e.g*., antibiotic resistance and environmental pollutants of its residues), the use of antibiotics has been restricted worldwide, which results in the urgent need to find alternative routes to manage animal health and maintain production efficiency (50, 51). *Lactobacillus* species are one of the most commonly used probiotic agents in swine and are considered one such potent AGPs replacement (18, 52, 53). Therefore, this study systematically reviewed and performed a set of meta-analyses to determine the effects of probiotic *Lactobacillus* spp. on porcine growth performance and intestinal morphology. The main findings were as follows: (1) *Lactobacillus* spp. supplementation can improve piglets' performance including ADFI, ADG and the G/F ratio, and is superior to antibiotics in growth promotion; (2) *Lactobacillus* spp. supplementation substantially modified the small intestinal morphology, increased VH and the V/C ratio of piglets in all segments, whereas decreased the jejunal and ileal CD.

The growth performance is an essential indicator of pig health and the economic benchmark of the production system. Comparable to the AGPs, there are also theories on the mechanism of growth improvement of probiotics in animals, which include improving the gut barrier function, nutrient utilization, gut microbiome, intestinal morphology, and immunity (18). In particular, important indexes like average daily gain, average daily feed intake, and the gain-to-feed ratio reflect nutrient uptake and absorptive capacity, where higher values in the *Lactobacillus*-treated piglets implied improved nutrient utilization (45). Our meta-analysis revealed that ADG and ADFI were increased by 13.8% and 7.02% on average by *Lactobacillus* addition. It is suggested that *Lactobacillus-*based feeding could enhance feed palatability (54), which may explain the increased feed intake. A probiotic*-*improved G/F ratio was reported by us, suggesting that *Lactobacillus* supplementation brings about a more cost-effective feeding program than the control piglets. Another mechanism by which *Lactobacillus* spp. enhance animal performance may be *via* the promotion of beneficial bacteria and inhibition of harmful microorganisms in the intestinal microenvironment (55).

Growing evidence indicates that gut microbiota plays a crucial role in host metabolic health and fitness. A healthy small intestine has a dominance of *Lactobacillus* spp., which may be disrupted by perturbations like weaning and changes of feed in piglets (56). By adding a *Lactobacilli* compound (including *L. gasseri, L. reuteri, L. acidophilus* and *L. fermentum*), Huang et al. showed that significantly decreased the *E. coli* and aerobe counts, and increased *Lactobacilli* and anaerobe counts in the digesta and mucosa, thereby promoting growth performance of pigs (57). Furthermore, *Lactobacillus* spp. can increase the levels of microbial metabolites such as butyrate to alleviate piglet diarrhea, which directly and indirectly affects growth performance (58). Although the gut microbiome is not analyzed here, we choose to study the effects of *Lactobacillus* spp. in piglets at the age of weaning. When antibiotics are often used due to sudden changes in diets and gut microbiota dysbiosis, which further impair pig performance and health. It is noteworthy that at weaning, a reduction of intestinal VH or villus atrophy may occur (56).

In this regard, we found that *Lactobacillus* spp. supplementation significantly increased VH and the V/C ratio in the small intestine of piglets. It may be partly due to the *Lactobacillus*-increased daily feed intake, resulting in a trophic action on the development of intestinal epithelium (18). As the main digestive and absorptive site, increases in small intestinal VH and the V/C ratio are directly related to the larger surface area and enhanced epithelium turnover and cell mitosis activation (24, 59). It allows for enhanced uptake of dietary substances while excluding noxious agents in the gut lumen (60). Finally, the improved gut morphology can facilitate digestion, and absorption of nutrients, fluid, and electrolytes for piglets (18, 24, 59, 60), promoting growth performance (61). This was supported by studies we have summarized in our meta-analysis and numerous other studies (14, 18). In addition, in piglets challenged with LPS or *E. coli*, carbohydrate and fatty acid utilization can be compromised due to inflammation, while the probiotic can alter the villus-crypt architecture and influence the associated enzyme activity and nutrient transport receptor expression (62, 63). Similarly, Zhang et al. demonstrated that *Lactobacilli* supplementation increases digestive enzyme activities and promotes growth performance (12). Furthermore, the addition of *Lactobacillus* resulted in the enhancement of genes for the metabolism, transport, and catabolism of vitamins, amino acids, lipids, and polyketides, thus the improvement of growth performance (64). In addition, we also reported a location-specific response of intestinal histology driven by *Lactobacillus* spp. *i.e., Lactobacillus* supplementation improved all jejunal and ileal histological parameters, likely related to the gradient distribution of microbiota along the pig small intestine (65, 66). It is also suggested that the probiotics, live bacteria, their signaling or metabolites, must have reached the distal part of the intestine in piglets and become effective. However, care must be taken as the meta-analysis approach has limitations. For instance, when the number of included studies is small, the number of trial characteristics is large, and the heterogeneity of data becomes large, which was also seen in our studies. And even if the number of studies is increased, Meta-analysis might not fully explain all but kept a residual heterogeneity (21, 22, 25).

## Conclusion

In conclusion, our findings indicate that *Lactobacillus* spp. supplementation plays a crucial role in improving growth performance of piglets by increasing ADF, ADFI and the G/F ratio, in parallel modifying the intestinal morphology, especially in the jejunum and ileum. It suggests that *Lactobacillus* spp. can be regarded as a promising alternative to replace AGPs usage in pig production. Based on our analysis, we suggest that future studies focus on documenting the effects of *Lactobacillus* spp. supplementation on the porcine gut microbiome; evaluating probiotics viability in farm conditions and generating protocols and regulations for the application in the industry.

## Data availability statement

The original contributions presented in the study are included in the article/supplementary material, further inquiries can be directed to the corresponding authors.

## Author contributions

PH and H-YL: conceptualization, writing, and supervision. CuZ and JY: formal analysis. CuZ, JY, MZ, ChZ, LY, and ZL: investigation. CuZ, JY, PH, and H-YL: writing the original draft. DC and SC: reviewing, revising, and editing the manuscript. DC, PH, and H-YL: funding acquisition. All authors contributed to the article and approved the submitted version.

## Funding

This work was supported by the Postgraduate Research and Practice Innovation Program of Yangzhou University (X20211025), the Natural Science Foundation of Jiangsu Province (BK20220582), and the Priority Academic Program Development of Jiangsu Higher Education Institutions (PAPD).

## Conflict of interest

The authors declare that the research was conducted in the absence of any commercial or financial relationships that could be construed as a potential conflict of interest.

## Publisher's note

All claims expressed in this article are solely those of the authors and do not necessarily represent those of their affiliated organizations, or those of the publisher, the editors and the reviewers. Any product that may be evaluated in this article, or claim that may be made by its manufacturer, is not guaranteed or endorsed by the publisher.

## References

[1] Pedroso AA Menten JFM Lambais MR Racanicci AMC Longo FA Sorbara JOB. Intestinal bacterial community and growth performance of chickens fed diets containing antibiotics. Poult Sci. (2006) 85:747–52. 10.1093/ps/85.4.747 16615359

[2] Dibner JJ Richards JD. Antibiotic growth promoters in agriculture: history and mode of action. Poult Sci. (2005) 84:634–43. 10.1093/ps/84.4.634 15844822

[3] Luecke RW Thorp F. Jr., Newland HW, Mc MW. The growth promoting effects of various antibiotics on pigs. J Anim Sci. (1951) 10:538–42. 10.2527/jas1951.102538x 14832161

[4] Rodrigues G Maximiano MR Franco OL. Antimicrobial peptides used as growth promoters in livestock production. Appl Microbiol Biot. (2021) 105:7115–21. 10.1007/s00253-021-11540-3 34499200

[5] Li J. Current status and prospects for in-feed antibiotics in the different stages of pork production - a review. Asian-Australas J Anim Sci. (2017) 30:1667–73. 10.5713/ajas.17.0418 28823126 PMC5666167

[6] Wang S Zeng X Yang Q Qiao S. Antimicrobial peptides as potential alternatives to antibiotics in food animal industry. Int J Mol Sci. (2016) 17:7050603. 10.3390/ijms17050603 27153059 PMC4881439

[7] Liu YY Wang Y Walsh TR Yi LX Zhang R Spencer J. Emergence of plasmid-mediated colistin resistance mechanism MCR-1 in animals and human beings in China: a microbiological and molecular biological study. Lancet Infect Dis. (2016) 16:161–8. 10.1016/S1473-3099(15)00424-7 26603172

[8] E.C. (EC). Ban on Antibiotics as Growth Promoters in Animal Feed Enters into Effect. (2005). Available online at: http://europa.eu/rapid/press-release_IP-05-1687_en.html (accessed 13, September 2018).

[9] C.f.D.C.a.P. (CDC), Food and Food Animals. (2020). Available online at: https://www.cdc.gov/drugresistance/food.html (accessed 18, June 2020).

[10] Ministry of Agriculture and Rural Affairs of the People's Republic of China 2018. Notice of the Ministry of Agriculture and Rural Affairs Office on the Pilot Work on the Reduction of the Use of Veterinary Antimicrobial Drugs. (2018). Available online at: http://www.moa.gov.cn/govpublic/SYJ/201804/t20180420_6140711.htm (accessed May 27, 2019).

[11] Salminen S Collado MC Endo A Hill C Lebeer S Quigley EMM. The international scientific association of probiotics and prebiotics (ISAPP) consensus statement on the definition and scope of postbiotics. Nat Rev Gastroenterol Hepatol. (2021) 18:649–67. 10.1038/s41575-021-00440-6 33948025 PMC8387231

[12] Zhao PY Kim IH. Effect of direct-fed microbial on growth performance, nutrient digestibility, fecal noxious gas emission, fecal microbial flora and diarrhea score in weanling pigs. Anim Feed Sci Tech. (2015) 200:86–92. 10.1016/j.anifeedsci.2014.12.010

[13] Yu Q Yuan L Deng J Yang Q. *Lactobacillus* protects the integrity of intestinal epithelial barrier damaged by pathogenic bacteria. Front Cell Infect Microbiol. (2015) 5:26. 10.3389/fcimb.2015.00026 25859435 PMC4373387

[14] Sayan H Assavacheep P Angkanaporn K Assavacheep A. Effect of *Lactobacillus salivarius* on growth performance, diarrhea incidence, fecal bacterial population and intestinal morphology of suckling pigs challenged with F4+ enterotoxigenic escherichia coli. Asian Austral J Anim Sci. (2018) 31:1308–14. 10.5713/ajas.17.0746 29642683 PMC6043459

[15] Yi H Wang L Xiong Y Wen X Wang Z Yang X. Effects of *Lactobacillus reuteri* LR1 on the growth performance, intestinal morphology, and intestinal barrier function in weaned pigs. J Anim Sci. (2018) 96:2342–51. 10.1093/jas/sky129 29659876 PMC6095392

[16] Wang TW Teng KL Liu YY Shi WX Zhang J Dong EQ. *Lactobacillus plantarum* PFM 105 promotes intestinal development through modulation of gut microbiota in weaning piglets. Front Microbiol. (2019) 10:90. 10.3389/fmicb.2019.00090 30804899 PMC6371750

[17] Liu H Dicksved J Lundh T Lindberg JE. Heat shock proteins: intestinal gatekeepers that are influenced by dietary components and the gut microbiota. Pathogens. (2014) 3:187–210. 10.3390/pathogens3010187 25437614 PMC4235725

[18] Dowarah R Verma AK Agarwal N. The use of *Lactobacillus* as an alternative of antibiotic growth promoters in pigs: a review. Anim Nutr. (2017) 3:1–6. 10.1016/j.aninu.2016.11.002 29767055 PMC5941084

[19] Wang Y Gong L Wu YP Cui ZW Wang YQ Huang Y. Oral administration of *Lactobacillus rhamnosus* GG to newborn piglets augments gut barrier function in pre-weaning piglets. J Zhejiang Univ Sci B. (2019) 20:180–92. 10.1631/jzus.B1800022 30666850 PMC6381001

[20] Patil AK Kumar S Verma AK Baghel RPS editors. Probiotics as feed additives in weaned pigs: a review. Livest Res Int. (2015) 3:31–9.

[21] Ones DS Viswesvaran C Schmidt FL. Realizing the full potential of psychometric meta-analysis for a cumulative science and practice of human resource management. Hum Resour Manag Rev. (2017) 27:201–15. 10.1016/j.hrmr.2016.09.011

[22] Pigott T. Methods of meta-analysis: correcting error and bias in research findings. Eval Program Plann. (2006) 29:236–7. 10.1016/j.evalprogplan.2006.06.002

[23] Bailey M. The mucosal immune system: recent developments and future directions in the pig. Dev Comp Immunol. (2009) 33:375–83. 10.1016/j.dci.2008.07.003 18760299

[24] Rieger J. The intestinal mucosal network in the pig: a histological view on nutrition-microbiota-pathogen-host-interactions. Veterinärmedizin. (2016) 569:641–8.

[25] Brooke BS Schwartz TA Pawlik TM MOOSE. reporting guidelines for meta-analyses of observational studies. JAMA Surg. (2021) 156:787–8. 10.1001/jamasurg.2021.0522 33825847

[26] Xu BC Fu J Zhu LY Li Z Wang YZ Jin ML. Overall assessment of antimicrobial peptides in piglets: a set of meta-analyses. Animal. (2020) 14:2463–71. 10.1017/S1751731120001640 32635952

[27] Guyot P Ades AE Ouwens MJNM Welton NJ. Enhanced secondary analysis of survival data: reconstructing the data from published Kaplan-Meier survival curves. BMC Med Res Methodol. (2012) 12:9. 10.1186/1471-2288-12-9 22297116 PMC3313891

[28] Suo C Yin Y Wang X Lou X Song D Wang X. Effects of *Lactobacillus plantarum* ZJ316 on pig growth and pork quality. BMC Vet Res. (2012) 8:89. 10.1186/1746-6148-8-89 22731747 PMC3482153

[29] Chen FM Wang HJ Chen JY Liu Y Wen W Li YH. *Lactobacillus delbrueckii* ameliorates intestinal integrity and antioxidant ability in weaned piglets after a lipopolysaccharide challenge. Oxid Med Cell Longev. (2020) 2020:6028606. 10.1155/2020/6028606 32104535 PMC7035547

[30] Liu H Zhang J Zhang SH Yang FJ Thacker PA Zhang GL. Oral administration of *Lactobacillus fermentum* I5007 favors intestinal development and alters the intestinal microbiota in formula-fed piglets. J Agr Food Chem. (2014) 62:860–6. 10.1021/jf403288r 24404892

[31] Yang KM Jiang ZY Zheng CT Wang L Yang XF. Effect of *Lactobacillus plantarum* on diarrhea and intestinal barrier function of young piglets challenged with *Enterotoxigenic Escherichia coli* K88. J Anim Sci. (2014) 92:1496–503. 10.2527/jas.2013-6619 24492550

[32] Wang Q Sun Q Qi RL Wang J Qiu XY Liu ZH. Effects of *Lactobacillus plantarum* on the intestinal morphology, intestinal barrier function and microbiota composition of suckling piglets. J Anim Physiol Anim Nutr (Berl). (2019) 103:1908–18. 10.1111/jpn.13198 31498508

[33] Qiao J Li H Wang Z Wang W. Effects of *Lactobacillus acidophilus* dietary supplementation on the performance, intestinal barrier function, rectal microflora and serum immune function in weaned piglets challenged with *Escherichia coli* lipopolysaccharide. Antonie Leeuwenhoek. (2015) 107:883–91. 10.1007/s10482-015-0380-z 25577203

[34] Lan R Koo J Kim I. Effects of *Lactobacillus acidophilus* supplementation on growth performance, nutrient digestibility, fecal microbial and noxious gas emission in weaning pigs. J Sci Food Agric. (2017) 97:1310–5. 10.1002/jsfa.7866 27342084

[35] Lee JS Awji EG Lee SJ Tassew DD Park YB Park KS. Effect of *Lactobacillus plantarum* CJLP243 on the growth performance and cytokine response of weaning pigs challenged with *Enterotoxigenic Escherichia* coli. J Anim Sci. (2012) 90:3709–17. 10.2527/jas.2011-4434 22859771

[36] Li YS San Andres JV Trenhaile-Grannemann MD van Sambeek DM Moore KC Winkel SM. Effects of mannan oligosaccharides and *Lactobacillus mucosae* on growth performance, immune response, and gut health of weanling pigs challenged with *Escherichia coli* lipopolysaccharides. J Anim Sci. (2021) 99:skab286. 10.1093/jas/skab286 34879142 PMC8653942

[37] Tang QS Yi HB Hong WB Wu QW Yang XF Hu SL. Comparative effects of *L. plantarum* CGMCC 1258 and *L. reuteri* LR1 on growth performance, antioxidant function, and intestinal immunity in weaned pigs. Front Vet Sci. (2021) 8:3389. 10.3389/fvets.2021.728849 34859082 PMC8632148

[38] Tian ZM Cui YY Lu HJ Ma XY. Effects of long-term feeding diets supplemented with *Lactobacillus reuteri* 1 on growth performance, digestive and absorptive function of the small intestine in pigs. J Funct Foods. (2020) 71:104010. 10.1016/j.jff.2020.104010

[39] Sonia T Ji H Hong-Seok M Chul-Ju Y. Evaluation of *Lactobacillus* and *Bacillus*-based probiotics as alternatives to antibiotics in enteric microbial challenged weaned piglets. Afr J Microbiol Res. (2014) 8:96–104. 10.5897/AJMR2013.6355

[40] Riboulet-Bisson E Sturme MH Jeffery IB O'Donnell MM Neville BA Forde BM. Effect of *Lactobacillus salivarius* bacteriocin Abp118 on the mouse and pig intestinal microbiota. PLoS One. (2012) 7:e31113. 10.1371/journal.pone.0031113 22363561 PMC3281923

[41] Liu H Ji HF Zhang DY Wang SX Wang J Shan DC. Effects of *Lactobacillus brevis* preparation on growth performance, fecal microflora and serum profile in weaned pigs. Livest Sci. (2015) 178:251–4.

[42] Moturi J Kim KY Hosseindoust A Lee JH Xuan B Park J. Effects of *Lactobacillus salivarius* isolated from feces of fast-growing pigs on intestinal microbiota and morphology of suckling piglets. Sci Rep. (2021) 11:6757. 10.1038/s41598-021-85630-7 33762614 PMC7990948

[43] Lee SI Kim HS Koo JM Kim IH. *Lactobacillus acidophilus* modulates inflammatory activity by regulating the TLR4 and NF-kappaB expression in porcine peripheral blood mononuclear cells after lipopolysaccharide challenge. Br J Nutr. (2016) 115:567–75. 10.1017/S0007114515004857 26769562

[44] Cao ST Wang L Jiao LF Lin FH Xiao K Hu CH. Effects of diosmectite-*Lactobacillus acidophilus* on growth performance, intestine microbiota, mucosal architecture of weaned pigs. Anim Feed Sci Tech. (2016) 220:180–6. 10.1016/j.anifeedsci.2016.08.012

[45] Jeong YD Ko HS Hosseindoust A Choi YH Chae BJ Yu DJ. *Lactobacillus*-based fermentation product and lactose level in the feed for weanling pigs: effects on intestinal morphology, microbiota, gas emission, and targeted intestinal coliforms. Livest Sci. (2019) 227:90–6. 10.1016/j.livsci.2019.06.018

[46] Liu H Ji HF Zhang DY Wang SX Wang J Shan DC. Effects of Lactobacillus brevis preparation on growth performance, fecal microflora and serum profile in weaned pigs. Livest Sci. (2015) 178:251–4. 10.1016/j.livsci.2015.06.002

[47] Lin L Chu H. Quantifying publication bias in meta-analysis. Biometrics. (2018) 74:785–94. 10.1111/biom.12817 29141096 PMC5953768

[48] Cox Laura M Yamanishi S Sohn J Alekseyenko Alexander V Leung Jacqueline M Cho I. Altering the intestinal microbiota during a critical developmental window has lasting metabolic consequences. Cell. (2014) 158:705–21. 10.1016/j.cell.2014.05.052 25126780 PMC4134513

[49] Gaskins HR Collier CT Anderson DB. Antibiotics as growth promotants: mode of action. Anim Biotechnol. (2002) 13:29–42. 10.1081/ABIO-120005768 12212942

[50] Tian M He XM Feng YZ Wang WT Chen HS Gong M. Pollution by antibiotics and antimicrobial resistance in livestock and poultry manure in China, and countermeasures. Antibiotics (Basel). (2021) 10:0050539. 10.3390/antibiotics10050539 34066587 PMC8148549

[51] Schulze M Nitsche-Melkus E Hensel B Jung M Jakop U. Antibiotics and their alternatives in artificial breeding in livestock. Anim Reprod Sci. (2020) 220:106284. 10.1016/j.anireprosci.2020.106284 32005501

[52] Hou CL Zeng XF Yang FJ Liu H Qiao SY. Study and use of the probiotic *Lactobacillus reuteri* in pigs: a review. J Anim Sci Biotechnol. (2015) 6:3. 10.1186/s40104-015-0014-3 25954504 PMC4423586

[53] Pupa P Apiwatsiri P Sirichokchatchawan W Pirarat N Maison T Koontanatechanon A. Use of *Lactobacillus plantarum* (strains 22F and 25F) and *Pediococcus acidilactici* (strain 72N) as replacements for antibiotic-growth promotants in pigs. Sci Rep. (2021) 11:5. 10.1038/s41598-021-91427-5 34103574 PMC8187408

[54] Cullen JT Lawlor PG Cormican P Gardiner GE. Microbial quality of liquid feed for pigs and its impact on the porcine gut microbiome. Animals (Basel). (2021) 11:2983. 10.3390/ani11102983 34680002 PMC8532943

[55] Dong XL Zhang NF Zhou M Tu Y Deng KD Diao QY. Effects of dietary probiotics on growth performance, faecal microbiota and serum profiles in weaned piglets. Anim Prod Sci. (2014) 54:616–21. 10.1071/AN12372

[56] Gresse R Chaucheyras-Durand F Fleury MA Van de Wiele T Forano E Blanquet-Diot S. Gut microbiota dysbiosis in postweaning piglets: understanding the keys to health. Trends Microbiol. (2017) 25:851–73. 10.1016/j.tim.2017.05.004 28602521

[57] Huang CH Qiao SY Li DF Piao XS Ren JP. Effects of *lactobacilli* on the performance, diarrhea incidence, VFA concentration and gastrointestinal microbial flora of weaning pigs. Asian Austral J Anim. (2004) 17:401–9. 10.5713/ajas.2004.401

[58] Yue Y He ZJ Zhou YH Ross RP Stanton C Zhao JX. *Lactobacillus plantarum* relieves diarrhea caused by enterotoxin-producing *Escherichia coli* through inflammation modulation and gut microbiota regulation. Food Funct. (2020) 11:10362–74. 10.1039/D0FO02670K 33220669

[59] Liao YL Jiang RL Lonnerdal B. Biochemical and molecular impacts of lactoferrin on small intestinal growth and development during early life. Biochem Cell Biol. (2012) 90:476–84. 10.1139/o11-075 22332905

[60] Kongsted H Jonach B Haugegaard S Angen Ø Jorsal SE Kokotovic B. Microbiological, pathological and histological findings in four danish pig herds affected by a new neonatal diarrhoea syndrome. BMC Vet Res. (2013) 9:206. 10.1186/1746-6148-9-206 24119974 PMC3852778

[61] Heo JM Opapeju FO Pluske JR Kim JC Hampson DJ Nyachoti CM. Gastrointestinal health and function in weaned pigs: a review of feeding strategies to control post-weaning diarrhoea without using in-feed antimicrobial compounds. J Anim Physiol Anim Nutr. (2013) 97:207–37. 10.1111/j.1439-0396.2012.01284.x 22416941

[62] Tsukahara T Inoue R Nakatani M Fukuta K Kishino E Ito T. Influence of weaning age on the villous height and disaccharidase activities in the porcine small intestine. Anim Sci J. (2016) 87:67–75. 10.1111/asj.12399 26153481

[63] Yin YB Guo SG Wan D Wu X Yin YL. Enteroids: promising *in vitro* models for studies of intestinal physiology and nutrition in farm animals. J Agr Food Chem. (2019) 67:2421–8. 10.1021/acs.jafc.8b06908 30739438

[64] Li P Zhou QQ Gu Q. Complete genome sequence of *Lactobacillus plantarum* LZ227, a potential probiotic strain producing B-group vitamins. J Biotechnol. (2016) 234:66–70. 10.1016/j.jbiotec.2016.07.020 27480344

[65] Wylensek D Hitch TCA Riedel T Afrizal A Kumar N Wortmann E. A collection of bacterial isolates from the pig intestine reveals functional and taxonomic diversity. Nat Commun. (2020) 11:6389. 10.1038/s41467-020-19929-w 33319778 PMC7738495

[66] Yang H Wu J Huang X Zhou Y Zhang Y Liu M. ABO genotype alters the gut microbiota by regulating GalNAc levels in pigs. Nature. (2022) 606:358–67. 10.1038/s41586-022-04769-z 35477154 PMC9157047

